# Prolonged systemic hyperglycemia does not cause pericyte loss and permeability at the mouse blood-brain barrier

**DOI:** 10.1038/s41598-018-35576-0

**Published:** 2018-11-29

**Authors:** Maarja Andaloussi Mäe, Tian Li, Giacomo Bertuzzi, Elisabeth Raschperger, Michael Vanlandewijck, Liqun He, Khayrun Nahar, Annika Dalheim, Jennifer J. Hofmann, Bàrbara Laviña, Annika Keller, Christer Betsholtz, Guillem Genové

**Affiliations:** 1 Department of Immunology, Genetics, and Pathology, Rudbeck Laboratory, Uppsala University, Uppsala, Sweden; 2 Integrated Cardio-Metabolic Centre, Department of Medicine, Karolinska Institute, Huddinge, Sweden; 3 University of Oxford; Physiology, Anatomy & Genetics, The Sherrington Building, Parks Road, Oxford, United Kingdom; 4 Department of Neurosurgery, Tianjin Medical University General Hospital, Tianjin Neurological Institute, Key Laboratory of Post-Neuroinjury, Neuro-repair and Regeneration in Central Nervous System, Ministry of Education and Tianjin City, Tianjin, China; 5 Department of Surgery, Cardinal Bernardin Cancer Center, Loyola University Chicago, Illinois, United States; 6 Concordia University, Austin, Texas United States; 7 Department of Neurosurgery, Clinical Neurocenter, Zürich University Hospital, Zürich University, Zürich, Switzerland

## Abstract

Diabetes mellitus is associated with cognitive impairment and various central nervous system pathologies such as stroke, vascular dementia, or Alzheimer’s disease. The exact pathophysiology of these conditions is poorly understood. Recent reports suggest that hyperglycemia causes cerebral microcirculation pathology and blood-brain barrier (BBB) dysfunction and leakage. The majority of these reports, however, are based on methods including *in vitro* BBB modeling or streptozotocin-induced diabetes in rodents, opening questions regarding the translation of the *in vitro* findings to the *in vivo* situation, and possible direct effects of streptozotocin on the brain vasculature. Here we used a genetic mouse model of hyperglycemia (*Ins2*^*AKITA*^) to address whether prolonged systemic hyperglycemia induces BBB dysfunction and leakage. We applied a variety of methodologies to carefully evaluate BBB function and cellular integrity *in vivo*, including the quantification and visualization of specific tracers and evaluation of transcriptional and morphological changes in the BBB and its supporting cellular components. These experiments did neither reveal altered BBB permeability nor morphological changes of the brain vasculature in hyperglycemic mice. We conclude that prolonged hyperglycemia does not lead to BBB dysfunction, and thus the cognitive impairment observed in diabetes may have other causes.

## Introduction

Chronic hyperglycemia constitutes the major systemic risk factor for microvascular complications in diabetes mellitus (DM), such as retinopathy^1^ and nephropathy^2^. Recent evidence also links hyperglycemia with cognitive deficits, as well as with central nervous system (CNS) pathologies including vascular dementia, stroke, and Alzheimer’s disease^3,4^. The underlying causes of hyperglycemia-induced CNS complications are poorly understood and likely multifactorial. It has been suggested that blood-brain barrier (BBB) damage may play a role^5^, a hypothesis supported by data from *in vitro* models of the BBB, as well as animal studies (reviewed in Prasad *et al*.^6^). However, modeling the full complexity of the BBB *in vitro* is currently not possible, weakening any extrapolation of the results to the *in vivo* situation. Also the animal studies, which were based on hyperglycemia induced by the beta cell toxin streptozotocin^7,8^, have caveats. Firstly, streptozotocin may not be entirely selective in its toxic activity; its effect could be broader^9^ and even directly target the brain vasculature. Secondly, hyperglycemia elicited by streptozotocin and similar drugs shows extensive inter-individual variability and depends on administration method, dosage, genetic background and other factors^10^. Finally, visualization and quantification of BBB permeability is still challenging, and different protocols may lead to different results. Therefore, whether hyperglycemia causes BBB dysfunction remains an open question.

The usage of genetic models of DM, such as the *Ins2*^*AKITA*^ mouse, constitutes a novel approach for studying hyperglycemia complications *in vivo*. The *Ins2*^*AKITA*^ mouse has a point mutation in the *Ins2* gene resulting in a conformational change in the protein that leads to its accumulation in pancreatic beta cells causing cell death^11^. This toxicity is completely specific to the *Ins2*-expressing beta cells, thus mimicking human type 1 DM. Heterozygous male *Ins2*^*AKITA*^ mice display a non-obese phenotype and develop consistent hyperglycemia, hypoinsulinemia, polydipsia, and polyuria around the age of 4 weeks^11^. Thus, the *Ins2*^*AKITA*^ mouse constitutes a clear advance over chemically-induced DM, as the primary insult is beta cell-specific. Additionally, this mouse model is stable and reproducible and hence allows longitudinal studies of hyperglycemia complications.

In our study, we sought to determine whether prolonged hyperglycemia causes BBB dysfunction and leakage in the mouse brain by using several state-of-the-art methodologies to characterize BBB dysfunction. We conducted our studies in *Ins2*^*AKITA*^ heterozygous males and littermate wild-type controls. The *Ins2*^*AKITA*^ mice yielded consistent, prolonged hyperglycemia throughout the study. BBB leakage was studied in long-term hyperglycemic animals by injecting different exogenous tracers intravenously^12^. BBB integrity was studied by quantifying and visualizing these injected tracers in the CNS^12,13^. Complementarily, we analyzed whether hyperglycemia leads to transcriptional or morphological changes in the mouse brain microvasculature. In sum, these methods failed to demonstrate increased BBB permeability in *Ins2*^*AKITA*^ mice.

Collectively, our results – along with the study by Corem and Ben-Zvi (submitted) - lead us to conclude that persistent systemic hyperglycemia *per se* does not cause BBB permeability in the mouse brain. In the light of novel studies in human DM patients showing an association with BBB leakage and dementia^14^, our study suggests that factors other than hyperglycemia contribute to BBB dysfunction.

## Material and Methods

### Animals

Male heterozygous *Ins2*^*AKITA*^ mice^11^ (referred here as *Ins2*^*AKITA*^) and littermate wild-type controls (referred here as WT) were used for this study. Mice were purchased from Jackson Labs (Bar Harbor, ME) on a C57Bl/6-J background and bred in house at the Scheele Animal House, Karolinska Institute. Animals were housed in Allentown XJ type II long cages (Allentown, NJ), with 12/12 h light-dark cycle and access to water and standard chow *ad libitum*. Cage bedding was changed 2–3 times per week due to polyuria. Glycemia (Bayer Contour, Solna, Sweden; range 0.6–33.3 mmol/l) and body weight were monitored weekly from 6 weeks of age. Mice were sacrificed between 26 to 38 weeks of age, and samples collected. All animals were sacrificed between 10–11 AM. The procedures were carried out in accordance with institutional and national Swedish policies following approval from the Animal Ethical Board of Northern Stockholm.

### Immunohistochemistry

Mice were perfused transcardially under full anesthesia (hypnorm-midazolam cocktail) with Hank’s Buffered Salt Solution (HBSS, cat. #14025092, Life Technologies, Sweden) followed by 4% paraformaldehyde (PFA) in Phosphate Buffered Saline (PBS). Tissues were removed and postfixed in 4% PFA in PBS for 4 h at 4 °C. Fifty μm brain sagittal vibratome sections were incubated in blocking/permeabilization solution (1% bovine serum albumin (BSA), 0.5% TritonX-100 (Sigma-Aldrich, St. Louis, MO) in PBS overnight at 4 °C, followed by incubation in primary antibody solution for 48 h at 4 °C, and subsequently in secondary antibody solution, overnight at 4 °C. Sections were mounted in Prolong Gold anti-fade reagent with DAPI (cat. #P36930, Thermo Fischer Scientific, Sweden). Primary antibodies used: goat anti-mouse PECAM1 (cat. #AF3628, R&D Systems, Abingdon, UK), rat anti-mouse Laminin α-2 chain (LAMA2) (cat. #Ab11576, Abcam, Cambridge, UK), rat anti-mouse ANPEP (cat. #MCA2183EL, AbD Serotec); rabbit anti-mouse Collagen IV (cat. #2150–1470, Bio-Rad Antibodies, Oxford, UK); rabbit anti-GLUT1 (cat. #07–1401, Millipore, Billerica, MA); rat anti-mouse PDGFR-β (cat. #14–1402, Thermo Fischer Scientific), rabbit anti-Vitronectin (VTN) (cat. #GWB-794F8F, Genway Biotech, San Diego, CA), rabbit anti-Desmin (DES) (cat. #Ab15200-1, Abcam), rat anti-GFAP (cat. #13-0300, Thermo Fischer Scientific), rabbit anti-AIF1 (cat. #019-19741, Wako, Neuss, Germany). Secondary antibodies for multiple labelling (donkey anti-rat, donkey anti-rabbit, donkey anti-goat) conjugated with the appropriate fluorescent dyes were from Thermo Fischer Scientific or Jackson ImmunoResearch (West Grove, PA). As a negative control the sections were incubated with secondary antibodies only. Image processing was done using Volocity 64 (Improvision, Coventry, UK), Fiji, Adobe Photoshop CS6, and Adobe Illustrator CS6 (Adobe Systems, San José, CA). All immunohistochemistry images were taken with Leica TCS SP8 confocal microscope (Leica Microsystems, Wetzlar, Germany) and presented as maximum intensity reconstructions of confocal z-stacks.

### Quantification of pericyte coverage

Pericyte coverage was quantified as previously reported^15^. Briefly, 3D confocal images were projected with maximum intensity projection from 10 μm thick z-stacks, contrast enhanced, de-speckled, smoothened, and a threshold was set to generate a binary image. These images were then subjected to ‘dilate’ and ‘close’ commands prior to running the AnalyzeSkeleton plug-in in Fiji (https://fiji.sc/). Total skeletal length was calculated for both PECAM1 stained images (all vessels) and ANPEP stained images (pericyte-covered vessels), and the ratio of the vessel lengths was used as a measurement of coverage. One to three fields per mouse were acquired with 20× objective from cerebral cortex of seven WT and six *Ins2*^*AKITA*^ mice. All image processing was done automatically with a custom macro designed in Fiji.

### Quantification of microglia processes

Three fields per mouse were acquired with 40× objective from cerebral cortex of six WT and seven *Ins2*^*AKITA*^ mice. Ten μm thick confocal stacks of AIF1 stained brain sections were analyzed with Imaris ×64 8.3.1 software (Bitplane, Belfast, UK). Microglia filament length in μm was measured in 3D-mode by filament module.

### Intravenous injection and detection of leakage tracers

To address BBB permeability, we injected intravenously either Evans Blue dye (EB, 2% in PBS, Sigma Aldrich, Saint Louis, MO) or lysine-fixable cadaverine conjugated to Alexa Fluor 488 or 555 (1 KDa, 5 mg/ml in 0.9% NaCl, Invitrogen). Circulation time was overnight for EB and 2 h for cadaverine. As a positive control for EB leakage we used *pdgfb*^*ret/ret*^ mice^12^. Animals were subsequently perfused transcardially with PBS for 5′ and brains removed. Successful cadaverine injections were verified by examining kidneys of injected animals under a fluorescence stereomicroscope (Leica Microsystems). Quantification of extravasated EB or cadaverine in the brain parenchyma was performed as published^12,16^.

### Microvasculature isolation for quantitative PCR and RNA sequencing

Purification of microvasculature was performed as described^17^ with modifications. Briefly, after collagenase A treatment, brain tissue was first gently pressed through a 100 μm and then through a 40 μm cell strainer. The tissue homogenate was incubated with rat anti-PECAM1 antibody-coated magnetic beads (Dynabeads, Invitrogen, cat. #11035) at 4 °C for 1 h. Microvascular fragments adherent to magnetic beads were washed 6 times with HBSS containing 1% BSA followed by 2 times with HBSS alone. Microvasculature fragments were lysed in 350 μl RLT buffer (Qiagen, Hilden, Germany).

### Quantitative PCR

Total RNA was isolated from whole cerebral cortex or purified brain microvasculature using RNeasy Mini kit (Qiagen). RNA was DNase-treated (Invitrogen) and samples were cleaned using RNeasy Mini-elute Cleanup Kit (Qiagen). cDNA was synthesized from 1 ug of total RNA using Superscript III (Invitrogen) and mRNA expression quantified by Taqman assays (Applied Biosystems) for mouse *Pdgfrb* (*Mm00435546_m1_FAM*), *Rgs5 (Mm00654112_m1)*, *Gfap (Mm01253033_FAM)* and mouse *Gapdh* (*Mm99999915_g1_FAM*) in the purified brain microvasculature samples and for mouse *Gfap (Mm01253033_FAM)* and mouse *Hprt (Mm03024075_m1_FAM)* in the cerebral cortex samples. Reference genes *Gapdh* and *Hprt* were used for ΔΔCt calculations. Gene of interest expression was presented as fold change over reference gene.

### RNA sequencing library preparation

RNA integrity and concentration of the isolated brain microvasculature was checked with the Agilent RNA 6000 pico kit and the Agilent 2100 Bioanalyzer (Agilent Biotechnologies, Santa Clara, CA). For conversion of the RNA to a cDNA library for Illumina sequencing, the SMARTer® Stranded Total RNA Sample Prep Kit - Low Input Mammalian kit was used according to the manufacturer’s protocol (Clontech, Mountain View, CA). The total RNA was depleted from rRNA and tRNA’s with the RiboGone- Mammalian kit (Clontech) prior to library construction.

### RNA sequencing data analysis

The RNA-seq libraries were prepared as previously described^18^. The samples were sequenced on an Illumina HiSeq. 2500 sequencer at Science for Life laboratory (SciLife Lab), Uppsala sequencing node. The sequence reads were mapped to the Ensembl mouse gene assembly (version: NCBIM37) using TopHat2 software (version 2.0.4)^19^, and the duplicated reads were filtered using the Picard tool (version 1.92, http://broadinstitute.github.io/picard/). Statistical tests were performed using the Cufflinks tool (version 2.2.1)^20^. Gene expression levels in brain microvasculature between the *Ins2*^*AKITA*^ and WT mice were compared, and the genes with multiple-test corrected *p* < 0.05 were identified as significantly differentially expressed genes. The sequence data is deposited in NCBI GEO, accession number GSE113919.

### Statistical analysis

Data are presented as mean ± SEM. For two group comparisons two-tailed, unpaired student’s *t* test was performed (Prism v5.01, GraphPad, La Jolla, CA). A p-value of ≤0.05 was considered statistically significant.

## Results

### The effect of hyperglycemia on blood-brain barrier permeability

**Figure 1:**
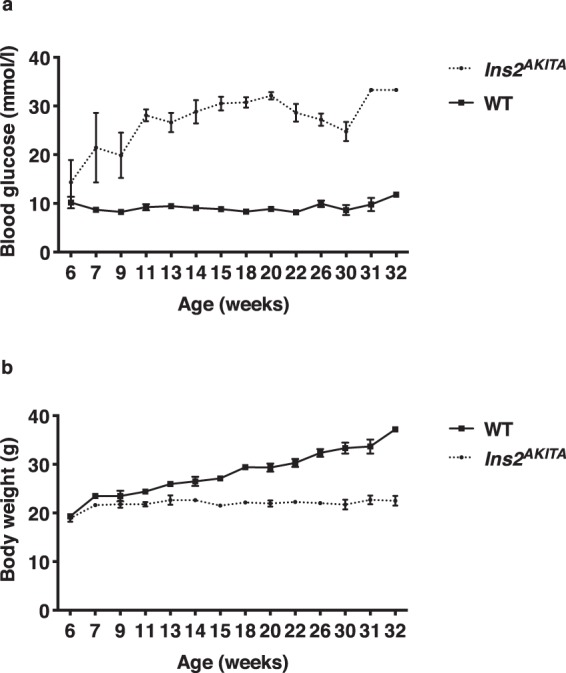
(**a**) Fasting blood glucose levels (mmol/l) and (**b**) body weight (**g**) of male *Ins2*^*AKITA*^ and WT littermate controls. Data is presented as mean ± SEM. n = 2 to 15 mice.

We first checked blood glucose levels in WT and *Ins2*^*AKITA*^ mice. As reported elsewhere^21–23^, *Ins2*^*AKITA*^ animals had elevated glycemia at 6 weeks of age as compared to WT mice (Fig. 1a). By 11 weeks, *Ins2*^*AKITA*^ animals were consistently hyperglycemic (>20 mmol/l), whereas WT mice remained normoglycemic (<10 mmol/l) throughout the experiment (Fig. 1a). Due to hypoinsulinemia, body weight in *Ins2*^*AKITA*^ mice reached a plateau by 7 weeks of age (≈20 g), while WT animals continued to gain weight normally (Fig. 1b).

**Figure 2:**
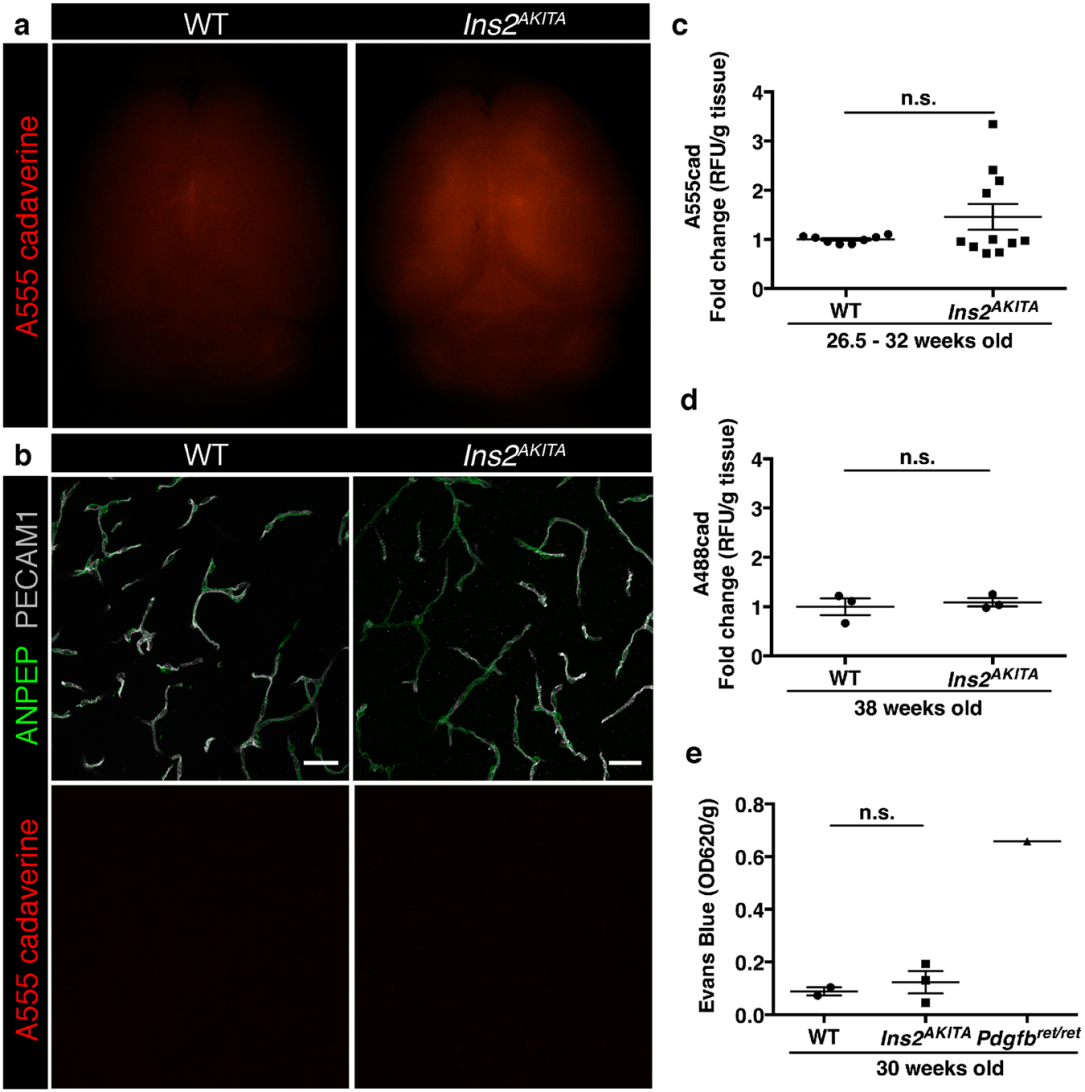
Blood-brain barrier permeability measurements in male *Ins2*^*AKITA*^ and WT littermate controls. (**a**) Representative stereomicroscope fluorescence images of brains showing 1 kDa Alexa Fluor 555 cadaverine permeability in *Ins2*^*AKITA*^ and WT after 2 h of dye circulation (n = 2). (**b**) Representative confocal images of coronal sections of 1 kDa Alexa Fluor 555 cadaverine injected mouse brains. ANPEP positive mural cells, green; PECAM1 positive vasculature, white. No Alexa Fluor 555 cadaverine leakage into brain parenchyma was observed either in *Ins2*^*AKITA*^ or WT mice (n = 2, scale bar 30 μm). (**c**) Quantification of 1 kDa Alexa Fluor 555 cadaverine permeability in 26.5–32 week-old *Ins2*^*AKITA*^ and WT mice after 2 h circulation (n = > 8, 3 independent experiments). y-axis shows the fold change in relative fluorescence units (RFU) per gram of brain tissue in relation to WT. (**d**) Quantification of 1 kDa Alexa Fluor 488 cadaverine permeability in 38 week-old *Ins2*^*AKITA*^ and WT mice after 2 h circulation (n = 3). y-axis shows the fold change in relative fluorescence units (RFU) per gram of brain tissue in relation to WT. (**e**) Evans Blue dye permeability in 30 week-old Ins2^AKITA^ and WT littermate control mice after overnight circulation (n = > 2). y-axis shows optical density (OD) at 620 nm per gram of tissue. *Pdgfb*^*Ret/Ret*^ served as positive control for tracer leakage into the brain parenchyma. n.s., not significant, student’s *t* test. Data is presented as mean ± SEM.

We then sought to address the effect of prolonged hyperglycemia on BBB permeability. We used two well-established methods to quantify vascular permeability in brain tissue^12^. Firstly, we injected the fluorescent tracer cadaverine-Alexa Fluor 488 or 555 (1 kDa) intravenously and perfused the animals after the dye had been circulating for 2 h. We could not detect a significant difference in tracer accumulation in brains between *Ins2*^*AKITA*^ and WT mice, either by microscopical visualization (Fig. 2a,b) or by fluorescence quantification (Fig. 2c). Additional analyses of BBB permeability in older mice (38 weeks of age) also failed to detect increased cadaverine extravasation into the brain of hyperglycemic mice (Fig. 2d). Secondly, we investigated whether larger molecular weight tracer and longer circulation time could reveal a compromised BBB function upon hyperglycemia. Thus, we intravenously injected the azo dye Evans Blue, which binds to albumin in the circulation, and allowed it to circulate overnight. As shown in Fig. 2e, quantification of Evans Blue accumulation in the brain was not significantly different between *Ins2*^*AKITA*^ and WT mice. As a positive control for BBB permeability, we quantified Evan’s blue extravasation in *Pdgfb*^*ret/ret*^ mice, a model of pericyte deficiency that reproducibly shows tracer leakage into the brain^12^ (Fig. 2e). The results obtained by these analyses lead us to conclude that long-term hyperglycemia does not cause any significant vascular leakage in the mouse brain.

### Study of pericyte coverage of *Ins2*^*AKITA*^ and WT brain capillaries

**Figure 3:**
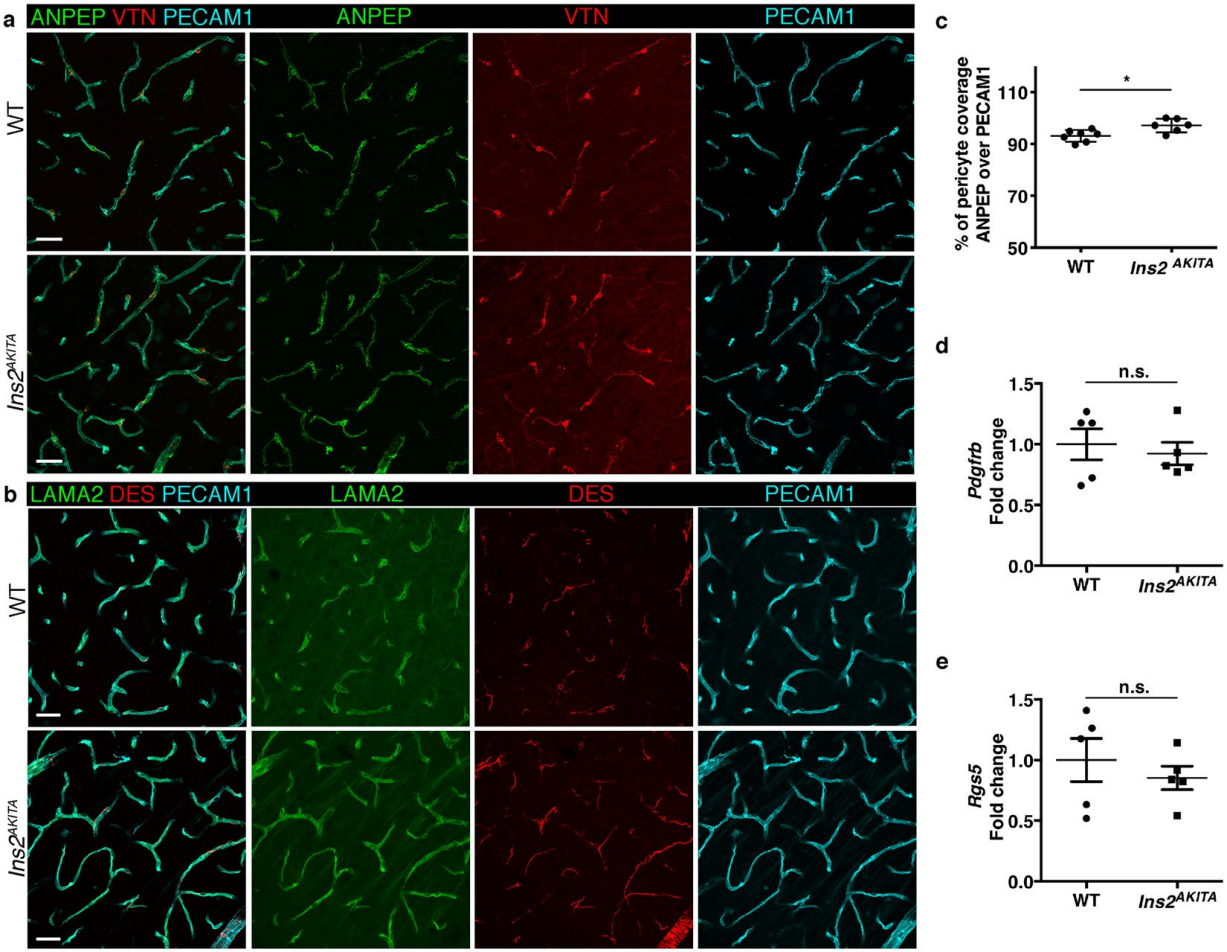
Characterization of pericyte coverage and pericyte-specific gene and protein expression in 26.5–32 week-old *Ins2*^*AKITA*^ and WT littermate controls. (**a,b**) Representative images of pericyte-specific protein expression in WT and *Ins2*^*AKITA*^ mice, (**a**) Aminopeptidase N (ANPEP, green) and Vitronectin (VTN, red); (**b**) Laminin alpha 2 (LAMA2, green) and desmin (DES, red). Endothelium visualized with PECAM1, cyan. n = 2, scale bar 30 μm. (**c**) The skeletal length of PECAM1 positive capillaries and ANPEP positive pericytes in *Ins2*^*AKITA*^ and WT was measured and plotted as the percentage of the pericyte length over vessel length (n = 6, *p = 0.0125, student’s *t* test). (**d,e**) qPCR analysis on isolated brain microvasculature fragments for pericyte-specific gene *Pdgfrb* (d) and *Rgs5* (**e**). WT controls are set as 1 and *Ins2*^*AKITA*^ results are presented as fold change over WT (n = 5). n.s. = not significant, student’s *t* test. Data is presented as mean ± SEM.

Pericyte loss is one of the earliest events in diabetic retinopathy^24,25^, therefore we sought to analyze pericyte coverage of brain capillaries in hyperglycemic and WT mice. First, we analyzed pericyte coverage by immunohistochemistry (Fig. 3a,b). We stained endothelial cells by PECAM1 and pericytes by ANPEP and DES. We also analyzed the abundance of pericyte-derived extracellular matrix proteins VTN and LAMA2^12,18^. These immunostainings did not reveal any vascular abnormalities or aberrant pericyte coverage of brain capillaries from both *Ins2*^*AKITA*^ and WT mice (Fig. 3a,b). Furthermore, none of the four pericyte markers analyzed displayed abnormal expression as judged by their pattern of immunoreactivity (Fig. 3a,b). Unexpectedly, pericyte coverage, quantified as the percentage of the PECAM1^+^ endothelium covered by ANPEP^+^ pericytes, was significantly increased in *Ins2*^*AKITA*^ as compared to WT (Fig. 3c).

We then obtained mRNA from isolated microvascular fragments, containing endothelial cells, pericytes, and astrocyte end-feet^17^. In agreement with our immunohistochemistry results, we did not detect differences in mRNA expression of pericyte-specific genes (*Pdgfrb* and *Rgs5*) by quantitative, real-time PCR (Fig. 3d,e).

Collectively, these results suggest that hyperglycemia does not cause decreases in pericyte coverage or changes in pericyte-specific gene or protein expression in the mouse brain.

### Brain vascular transcriptome analysis

**Figure 4:**
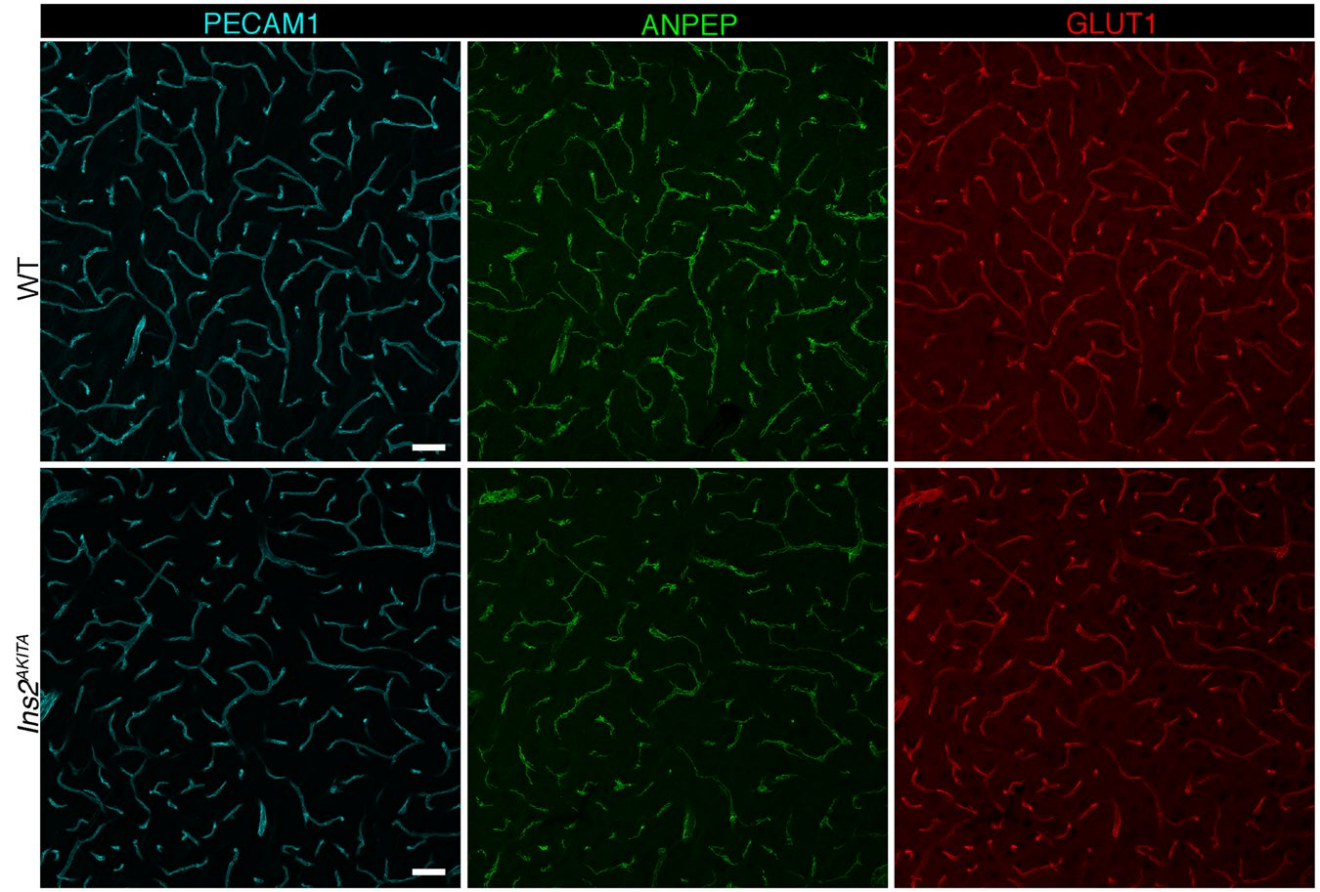
Representative image of immunostaining corresponding to the glucose transporter protein SLC2A1 (GLUT1) in 30 week-old *Ins2*^*AKITA*^ and WT cerebral cortex. Mural cells (ANPEP, green), glucose transporter 1 (GLUT1, red), endothelium (PECAM1, cyan). n = 2, scale bar 50 µm.

To take a more comprehensive effort in characterizing the brain vasculature upon hyperglycemia, we extracted mRNA from brain microvasculature fragments isolated from *Ins2*^*AKITA*^ and WT mice and analyzed their transcriptome by RNA sequencing (Table 1). Twenty-three genes were significantly changed in *Ins2*^*AKITA*^ as compared to WT. One gene, *Pin1*, was found to be upregulated in *Ins2*^*AKITA*^ mice, whereas the remaining 22 were downregulated. We checked the cellular origin of the 23 dysregulated genes according to the recently described molecular atlas of the mouse brain vasculature^13^. Only 4 genes were clearly associated with endothelial cell expression (Table 1), while the remaining 19 had a broad expression pattern spanning at least 2 different cell types. We did not find differentially regulated genes related to the tight junctions at the BBB, nor known genes mediating endothelium-pericyte interactions. Of relevance for the hyperglycemic status of the *Ins2*^*AKITA*^ mice, *Slc2a1* (*Glut1*) was 1.8-fold downregulated in *Ins2*^*AKITA*^ (Table 1). Immunohistochemistry analysis of SLC2A1 did not reveal an aberrant pattern of expression, however, a slightly weaker immunofluorescent staining was noticed in *Ins2*^*AKITA*^ as compared to WT (Fig. 4).

**Table 1:** RNA sequencing of microvascular fragments. Differentially expressed genes in *Ins2*^AKITA^ (n = 2) when compared to WT (n = 3) littermate controls. In the mouse brain, *Gata2, Sema3G, Slc2a1*, and *Gm694* displayed restricted endothelial expression.

Gene symbol	FPKM_WT	FPKM_Ins2^AKITA^	log2(Ins2^AKITA^/WT)	p_value
*Pin1*	9.54	21.42	1.17	5.00E-05
*Slc2a1*	191.65	105.16	−0.87	5.00E-05
*Akap8l*	66.40	35.47	−0.90	1.00E-04
*Miat*	57.07	28.77	−0.99	1.00E-04
*Col6a1*	30.61	15.40	−0.99	1.00E-04
*Rtl1*	36.01	18.07	−0.99	5.00E-05
*Net1*	40.09	20.02	−1.00	1.00E-04
*Snrnp70*	124.68	55.12	−1.18	5.00E-05
*Igsf9b*	22.51	9.66	−1.22	5.00E-05
*A930011O12Rik*	55.76	23.81	−1.23	5.00E-05
*Col11a1*	11.91	4.92	−1.28	5.00E-05
*Sik1*	16.27	6.69	−1.28	5.00E-05
*A230050P20Rik*	60.28	24.05	−1.33	5.00E-05
*Txnip*	55.65	21.76	−1.35	1.00E-04
*Usp36*	30.32	11.76	−1.37	5.00E-05
*Plat*	36.59	14.13	−1.37	5.00E-05
*Sema3g*	15.04	5.75	−1.39	5.00E-05
*Galntl2*	10.79	3.47	−1.64	1.00E-04
*Gata2*	19.73	6.13	−1.69	5.00E-05
*Stxbp2*	53.89	15.51	−1.80	5.00E-05
*Apex2*	16.89	3.32	−2.35	1.00E-04
*E2f6*	146.50	13.42	−3.45	5.00E-05
*Gm694*	9.75	0.00		5.00E-05

The rest of the genes had a broad expression pattern (FPKM, fragments per kilobase of exon per million reads mapped).

### Analysis of glial cells

**Figure 5:**
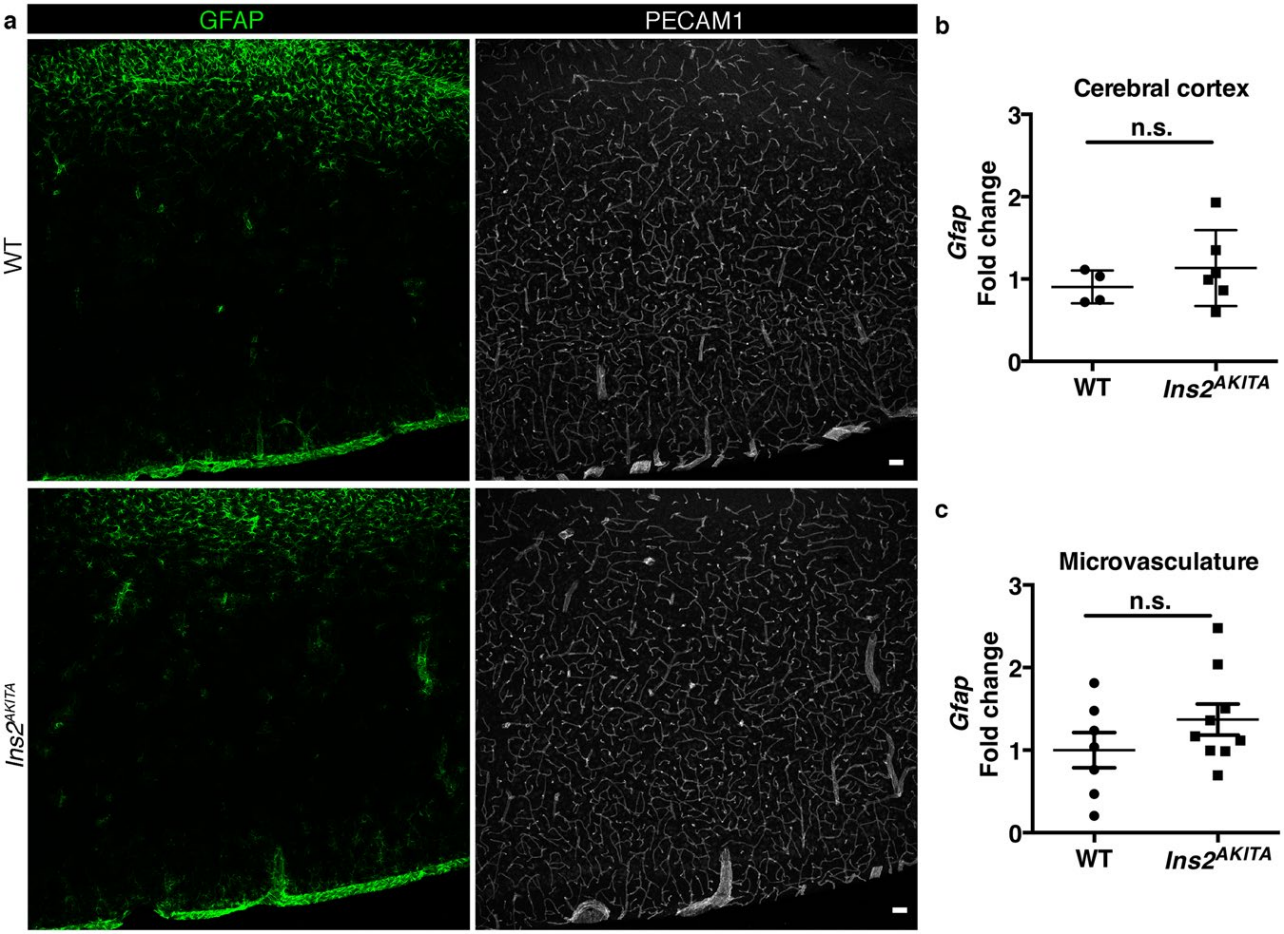
Characterization of reactive astrocytes in 26.5–32-week-old *Ins2*^*AKITA*^ and WT littermate controls. (**a**) Representative image of reactive astrocytes in *Ins2*^*AKITA*^ and WT cerebral cortex, astrocytes (GFAP, green), vasculature (PECAM1, white) (n = 5, scale bar 50 μm). (**b**) Total cerebral cortex *Gfap* mRNA expression in *Ins2*^*AKITA*^ (n = 6) and WT (n = 4) mice presented as fold change over *Hprt* reference gene expression. (**c**) *Gfap* mRNA expression from isolated cerebral microvascular fragments of *Ins2*^*AKITA*^ and WT mice presented as fold change over *Gapdh* reference gene (n = 7). n.s. = not significant, Student’s *t* test.

Hyperglycemia has been reported to cause early morphological and gene expression changes in glial cells, particularly in astrocytes and microglia^26^. We analyzed astrocytes by GFAP immunostaining. Additionally, we measured *Gfap* mRNA expression in the cerebral cortex homogenates and from isolated brain microvasculature fragments. GFAP immunohistochemistry did not reveal any differences between *Ins2*^*AKITA*^ and WT brains (Fig. 5a). *Gfap* mRNA expression was found to be unchanged in *Ins2*^*AKITA*^ mice when compared to WT (Fig. 5b,c). We conclude that hyperglycemia does not cause major changes in astrocytic GFAP expression in the mouse brain.

**Figure 6:**
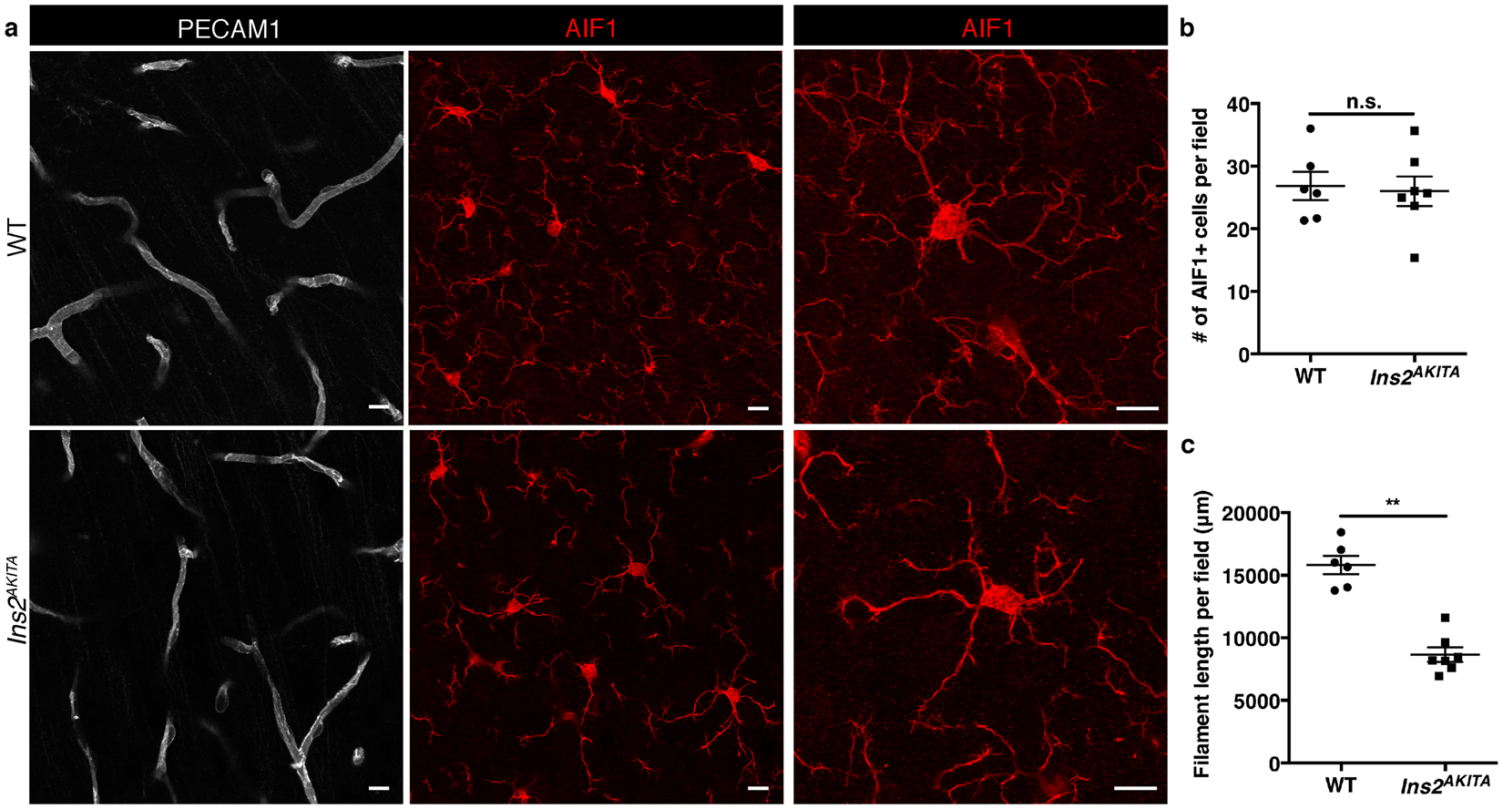
Characterization of microglia in 26.5–32 week-old *Ins2*^*AKITA*^ and WT littermate controls. (**a**) Representative images of microglia (AIF1, red) in *Ins2*^*AKITA*^ and WT cerebral cortex. Vasculature is stained with anti-PECAM1 antibody. Notice the fewer microglial processes in *Ins2*^*AKITA*^ cerebral cortex when compared to WT, scale bars 10 μm (n = 6). (**b**) Quantification of the number of AIF1 + microglia per field in WT and *Ins2*^*AKITA*^ cerebral cortex (n = 6), n.s. = not significant, student’s *t* test. (**c**) Quantification of the microglial filament length per field (μm) (n = 6), **p = 0.0012, student’s *t* test.

We then studied microglial cells by immunohistochemistry on brain sections from *Ins2*^*AKITA*^ and WT mice. AIF1^+^ immunoreactivity was readily detected (Fig. 6a), with no differences in AIF1^+^ cell numbers between *Ins2*^*AKITA*^ and WT mice (Fig. 6b). However, the morphology of the AIF1^+^ cells was remarkably different. In WT, the cell processes were long and highly ramified (Fig. 6a). In contrast, *Ins2*^*AKITA*^ AIF1^+^ cells had an apparent larger cell body and significantly shorter and less ramified processes (Fig. 6a,c). These morphological changes are consistent with the description of “activated” retinal microglia caused by hyperglycemia^23,26^.

## Discussion

The question whether hyperglycemia causes BBB permeability has been addressed in numerous reports^6^. Unfortunately, however, differences in experimental design and divergent and sometimes contradictory results between studies preclude consistent conclusions. This is not entirely surprising, however. The extremely delicate BBB architecture *in vivo*, including important features such as blood flow and its metabolic regulation (neurovascular coupling), makes it impossible to extrapolate *in vitro* results to the *in vivo* scenario. Previous animal studies are also problematic, since they applied chemically induced hyperglycemia^7,8^. Although popular, this technique has problems with specificity^9,27–29^ and variability as it depends on administration route, dosage, and genetic background^10^.

The advent of genetic mouse models, such as the *Ins2*^*AKITA*^ mouse^11^, offers the opportunity of studying the effect of hyperglycemia without many of the above-mentioned confounding factors. To date, a few reports have addressed the role of hyperglycemia on the blood-retinal barrier (BRB) in *Ins2*^*AKITA*^ mouse. Two studies found early signs of retinopathy including pericyte loss and increased BRB permeability^21,30^. Subsequently, it was shown that the presence of the *rd8* mutation in the *Crb1* gene – causing retinal degeneration^31^ - might account for the previous observations of advanced retinal pathology^23^. To the best of our knowledge, no studies of the BBB structure and function in *Ins2*^*AKITA*^ mice have previously been reported.

In the present study, we aimed at evaluating hyperglycemia-induced BBB permeability by employing highly specific tools. Firstly, a range of well-established techniques for the study of BBB and neurovascular unit (NVU) integrity^12,13^, and secondly, a robust mouse model of hyperglycemia, the *Ins2*^*AKITA*^, were used. Our data clearly show that prolonged systemic hyperglycemia does not elicit BBB dysfunction and leakage in the mouse brain. Moreover, different cellular and molecular components at the NVU appear intact in *Ins2*^*AKITA*^ mice, such as endothelial cells, pericytes, astrocytes, and basement membrane. Based on our results and the Corem and Ben-Zvi (submitted), we propose that hyperglycemia-induced BBB leakage is likely negligible.

Transcriptional analysis of microvascular fragments comparing hyperglycemic animals and WT controls revealed *Slc2a1* to be 1.8-fold downregulated in brain vessel-fragments from *Ins2*^*AKITA*^ animals (Table 1). Analysis of glucose transport in the brains of human patients has been controversial, with methods employed differing from surrogate tissues for SLC2A1 expression in the brain^32^ to transient hyperglycemia conditions in healthy human beings^33^. Others have shown a decrease in glucose uptake in experimental non-obese DM animals^34^. Our study shows that *Slc2a1* is downregulated at the transcriptional level specifically in the hyperglycemic mouse brain vasculature. Of note, allelic disorders associated with *SLC2A1* mutations, glucose-deficiency syndrome, epilepsy, and dystonia, are associated with cognitive disability^35^. Thus, downregulation of SLC2A1 on brain blood vessels due to hyperglycemia could lead to metabolic changes in neural tissue leading to cognitive dysfunction especially after repeated hypoglycemic episodes.

Interestingly, the only gene that was found to be upregulated in *Ins2*^*AKITA*^ mice was *Pin1*, a phospho-serine/threonine-proline isomerase that has been implicated in cellular proliferation. *Pin1* has previously been reported to be upregulated in an *in vitro* model of hyperglycemia and in the aorta of streptozotocin-induced diabetic mice, resulting in mitochondrial oxidative stress, vascular relaxation dysfunction and vascular inflammation^36^. Thus, our results imply that even if hyperglycemia-induced upregulation of *Pin1* might cause similar stresses to the brain vasculature, these do not induce significant BBB disruption.

Our analysis of microglia showed that these cells change their morphology in *Ins2*^*AKITA*^ mice into a more amoeboid-like shape with shorter and thicker processes compared to the highly ramified process morphology seen in WT brains. Our findings are consistent with an “activated” microglial response. How the microglia is activated is unknown, but it has been suggested that hyperglycemia can potentially activate retinal microglia through a variety of molecular mechanisms^37^. Furthermore, it is hypothesized that permeability at the BRB contributes to the microglia activation^37^. Our results reveal the same morphological changes in the hyperglycemic mouse brain than these already reported in retinas^23^. Because the integrity of the BBB and NVU are preserved in the *Ins2*^*AKITA*^ mice, other microglial activating factors than BBB permeability should be considered.

The association between DM and different brain pathologies is an underexplored but emerging research area. BBB dysfunction is commonly found in conditions such as Alzheimer’s disease, and it is therefore tempting to speculate that hyperglycemia-induced BBB permeability is the underlying mechanism of brain pathology in human DM patients^14^. However, even the most prevalent microvascular DM complications, such as retinopathy or nephropathy, do not affect all DM patients^38^. Beyond hyperglycemia, other DM features, such as hyperglycemia/hypoglycemia cycles, dyslipidemia, or genetic susceptibility should be considered as possible causal factors for these pathologies. Our results presented herein show that hyperglycemia has minor, if any, effect on BBB permeability, and therefore fails to support increased BBB leakage as an underlying cause of DM-related brain pathologies.

## Data Availability

The dataset generated and analyzed during the current study is available in NCBI GEO, accession number GSE113919 (https://www.ncbi.nlm.nih.gov/geo/query/acc.cgi?acc = GSE113919).
